# Problems faced by Researchers and pressure on Impact Factor Journal Editors

**DOI:** 10.12669/pjms.37.3.4296

**Published:** 2021

**Authors:** Shaukat Ali Jawaid

It is extremely painful not to accept a couple of manuscripts from researchers for further processing almost daily because of increased submissions and our financial as well as human resource constraints. At present we get almost about two hundred fifty submissions not only from Pakistan but from overseas from many countries in South East Asia and Asia-Pacific in particular every month while we have the facilities to process, manage and publish just about three hundred fifty to four hundred manuscripts every year. It leads to annoyance with authors whose papers are not accepted which is a real dilemma for the Editor. Editor colleagues of the other two Impact Factor medical journals published from Pakistan are faced with a similar situation.

While on one hand the authorities claim to promote research culture but at the same time the authors find it extremely difficult to get their studies published in good standard peer reviewed biomedical journals in Pakistan as they are too few just three. IF journals in Pakistan is their first preference to meet requirements of the regulatory bodies like Higher Education Commission which says that all PhD scholars and medical university faculty members should publish their work in Impact Factor Journals. Publication in IF journals overseas is very expensive which many authors cannot afford. Hence, the only way forward is to relax the rules and regulations by the regulatory bodies like HEC or include few more good quality medical journals in its list which even do not enjoy an Impact Factor at present but have applied, are included in the Journal Citation Report (JCR) and are hopeful of getting an IF in the coming years.

We on our part has time and again made it clear and highlighted the common reasons for not accepting manuscripts for further processing after initial screening and Editor’s Triage.^1^ Hence if the authors are careful, read the instructions for authors on our website and make sure that the problems and deficiencies highlighted earlier are avoided, it will minimize trauma to their manuscripts.^2^ To help authors we from time to time do include some manuscripts with the main objective of educating the authors.^3^

The situation can improve a lot if medical institutions organize regular training workshops for their faculty members, undergraduate and postgraduate students on Scientific Writing. Secondly each institution must have some facilities whereby the authors are given advice after looking at their manuscripts in pre-submission stage. The Dept. of Medical Education can also be entrusted this responsibility which will not only improve the quality of these manuscripts but also go a long way in reducing the rejection rate. At the same time all the journals editors should also feel it their responsibility to train the authors by facilitating training workshops in collaboration with different medical institutions. In Pakistan, a few distinguished medical editors have been facilitating such workshops all over the country from the platform of Pakistan Association of Medical Editors (PAME) for the last many years but still a lot more needs to be done.

**Fig.1:**
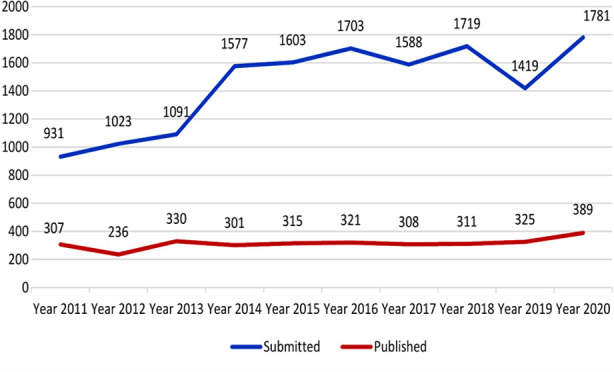
Submissions and acceptance Pak J Med Sci (2011-2020).

In order to know our strength and weaknesses, we started conducting our own publication audit a couple of years ago which is now done annually on regular basis. The last publication audit was done in 2019. 4 A careful analysis for the year 2020 revealed that we received 1,781 manuscripts after initial screening. Of these 389 were published after peer review, thirteen were rejected because of plagiarism, twenty eight were withdrawn by the authors themselves as they wanted quick publication soon after submission and we could not oblige them. Table-I. During the Year under review submissions from Pakistan were 854 and the rest were from overseas. Major submissions from overseas included 352 papers from Turkey, 202 from China, 144 from Saudi Arabia, 40 from Iran, 29 from Indonesia, 24 from Iraq and 14 from Egypt. Table-II. As compared to the previous year 2018 we receipted only 1419 submissions in 2019 since we were not entertaining new submissions for some time. New submissions were again stopped for three months during 2020 due to COVID19 Pandemic as our offices remained closed for some time and we wanted to clear the backlog first. However, despite that new submissions increased in 2020 to 1781 as compared to the previous year. Fig.1.

**Table-I:** PJMS manuscripts statistics of 2020 at a Glance.

Articles Published	389
Articles not accepted for further processing	1217
Articles Rejected because of Plagiarism	13
Articles Withdrawn by Authors	28
Under Process:	134
Total submissions	1781

**Table-II:** Country wise submissions during 2020.

Country	Total
Algeria	2
Australia	1
Bahrain	2
Canada	3
China	202
Colombia	2
Cyprus	3
Egypt	14
France	1
Georgia	1
Germany	1
India	10
Indonesia	29
Iran	40
Iraq	24
Ireland	3
Isle of Man	1
Italy	3
Jordan	9
Kazakhstan	3
Korea	7
Kuwait	2
Malaysia	9
Nigeria	11
Norway	3
Oman	1
**Pakistan**	**854**
Palestinian	1
Philippines	2
Portugal	1
Qatar	2
Russia	2
Saudi Arabia	144
Serbia	4
South Africa	2
Sudan	2
Thailand	3
Turkey	352
UAE	2
United Kingdom	12
United States of America	8
Viet Nam	1
Taiwan	1
Brazil	1

Grand Total	1781

Further analysis of submissions showed that the number of manuscripts published after peer review increased from 168 to 241 from Pakistan, from 46 to 66 from China, from 32 to 35 from Saudi Arabia. However, their number decreased from Turkey from 60 to 28, and four from eight from Islamic Republic of Iran. Table-III. One of the main reasons being that we encourage the authors from overseas to prefer publication of their research work in their own country’s standard peer reviewed journals in view of the increasing cost of publication charges.

**Table-III:** Manuscript published by Pak J Med Sci (2010 – 2020).

Country	2010	2011	2012	2013	2014	2015	2016	2017	2018	2019	2020
Algeria							1			1	
Australia							1	1	1	1	
Bahrain	1			1	1						
Bangladesh	4	4	4		1	1	1		1		
Canada											1
Cameroon	1										
China	1	18	34	80	89	65	66	54	43	46	66
Cyprus			1	1					3	1	
Fiji						1					
Germany				1							
India	2	1		1		1	1		1	1	
Iran	64	78	63	70	14	12	11	10	3	8	4
Iraq	1	3	2	3	1		1		2	3	1
Ireland											1
Japan								1			
Jordan	4		1							1	
Kenya			1								
Korea	1	2	1	3	2	5	7	2	2	1	
Kuwait	1		1								
Malaysia	1	9	3	9	7	10	6	1	1		2
Nigeria	10	9			4	4	1				1
Norway											1
Oman	1		1			1					1
Pakistan	56	93	65	91	93	106	135	163	150	168	241
Palestine	2			3	1	2					
Philippines							1				
Poland					2	4					
Romania				1	1	3	1	1	1		1
Saudi Arabia	11	6	16	17	19	32	23	21	24	32	35
Serbia								1	1	1	1
South Africa	3	2	1	6	2		1				1
Sudan	1										
Taiwan		2	2	3							
Thailand									2		
Turkey	34	74	37	38	60	65	63	49	72	60	28
UAE	4	1	1			2		1	3		1
Uganda			1								
UK	2	5	1	2		1	1	1	1	1	2
USA					4			2			1

Total	205	307	236	330	301	315	321	308	311	325	389

As expected majority of the submissions from within the country were again from major cities i.e. 261 from Karachi, 226 from Lahore, 101 from Islamabad, 62 from Peshawar, 43 from Rawalpindi, 24 from Faisalabad, 29 from Hyderabad including Jamshoro and 12 from Bahawalpur. Table-IV. Majority of the articles published during 2020 were also original manuscripts which accounted for 311 followed by Case Reports 13. Special and Brief or short communications accounted for 24, Editorials including Guest Editorials were ten. Table-V. We also published two special supplements during 2020 i.e. ICON 2020 in collaboration with Indus Health Network and to accommodate papers on Covid19 Pandemic, a special supplement which contained thirty three manuscripts was also published. Table-VI.

**Table-IV:** City wise submissions from Pakistan during 2020.

City	Total
Abbottabad	2
Azad Kashmir	2
Bahawalnagar	1
Bahawalpur	12
Bannu	1
Chakdara	1
Charsadda	2
Dera Ghazi Khan	3
Dera Ismail Khan	2
Faisalabad	24
Gujranwala	2
Gujrat	6
Haripur	2
Hyderabad	19
Islamabad	101
Jamshoro	10
Karachi	261
Khairpur	3
Kharian	1
Lahore	226
Larkana	2
Mansehra	3
Mardan	4
Mirpur, Azad Kashmir	1
Mirpurkhas	1
Multan	26
Nawabshah	1
Nowshera	1
Pakpattan	1
Peshawar	62
Quetta	11
Rahim Yar Khan	1
Rawalakot	1
Rawalpindi	43
Sahiwal	1
Sargodha	3
Sialkot	4
Swat	3
Taxila	2
Wah Cantt.	2

Grand Total	854

**Table-V:** Category Wise Manuscript Published in 2020.

Category	ICON 2020 Suppl	Jan-Feb 2020	Mar-Apr 2020	May-Jun 2020	Jul-Aug 2020	Sep-Oct 2020	Nov-Dec 2020	Covid-19 Suppl 2020	Grand Total
Acknowledgement	1	1							2
Book Review						1			1
Brief Communication						2	1	4	7
Case Reports	4	2	1	2	1	3			13
Clinical Case Series	1					1	1	1	4
Continuing Medical Education	1								1
Correspondence	2			1	1			1	5
Editorial	1	1	1		1	1			5
Guest Editorial	1		1	2	1			2	7
Guideline					1				1
Letter								2	2
Original Articles	11	54	51	44	45	46	50	10	311
Publication Audit				1					1
Review Article	1					1	1	4	7
Short Communication		2	2		1		2	2	9
Special Communications					1	1	2	4	8
Systematic Review				1	1				2
View Point								3	3

Grand Total	23	60	56	51	53	56	57	33	389

**Table-VI:** Country Wise Manuscript Published in 2020.

Country	ICON 2020 Suppl	Jan-Feb 2020	Mar-Apr 2020	May-Jun 2020	Jul-Aug 2020	Sep-Oct 2020	Nov-Dec 2020	COVID-19 Suppl. 2020	Grand Total
Canada						1			1
China		15	12	4	8	12	14	1	66
Iran		1		2			1		4
Iraq		1							1
Ireland							1		1
Malaysia		1				1			2
Nigeria					1				1
Norway							1		1
Oman					1				1
Pakistan	23	28	38	32	33	32	28	27	241
Romania			1						1
Saudi Arabia		5	4	8	4	4	7	3	35
Serbia							1		1
South Africa								1	1
Turkey		9	1	5	4	5	4		28
United Kingdom					1			1	2
United States of America						1			1
United Arab Emirates						1			1

Grand Total	23	60	56	51	53	56	57	33	389

Despite the fact that we always follow an author friendly policy, do our best to help, encourage and guide them but at times we find ourselves helpless when the authors do not bother to carefully read and follow the instructions for authors on the journal website, papers have numerous deficiencies or the English language and Grammar needs extensive improvement. We certainly cannot help re-write these papers which some of these submissions demand, hence the refusal to process them further.^5^

As a policy we prefer original research in clinical medicine. Manuscripts in certain disciplines like Dentistry, Physical Rehabilitation Medicine and Pharmacy as well as lengthy Reviews, basic sciences, Systemic Reviews and Meta-Analysis have a low priority with us though we do accommodate a few selected ones provided the topic is interesting. KAP studies, routine Survey reports and animal studies are generally not accepted due to our limitations. We feel that there is a great scope of good quality peer reviewed medical journals covering Basic Sciences, Allied Health Sciences including Nursing, Rehabilitation Medicine and Pharmacy. Let us hope that some people will come forward and fill this gap. In order to build the professional capacity of the editors, PAME in collaboration with University of Health Sciences Lahore has started a Certificate Course in Medical Editing.^6^ So far two batches have completed the training and the third batch will be inducted in May 2021. Hopefully it will also help the new editors thereby improving the quality of their journals.
